# Into the blue: the importance of murine *lacZ* gene expression profiling in understanding and treating human disease

**DOI:** 10.1242/dmm.023606

**Published:** 2015-11-01

**Authors:** Chris Armit

**Affiliations:** MRC Human Genetics Unit, Institute of Genetics and Molecular Medicine, University of Edinburgh, Edinburgh EH4 2XU, UK

**Keywords:** Gene expression, *lacZ*, Mouse

## Abstract

The International Mouse Phenotyping Consortium (IMPC) is a major international effort to explore the effects of knocking out 20,000 genes in the mouse. A new study by White and colleagues, published in the current issue of Disease Models & Mechanisms, demonstrates the usefulness of *lacZ in situ* reporter expression patterns in extending our understanding of genotype-phenotype relationships as part of the IMPC high-throughput screen. *In situ* gene expression profiling is invaluable for evaluating compartment-specific gene expression patterns, and these enrich our understanding of the role of genes in a great number of biological processes in multiple organ systems. Furthermore, the complexity of gene expression patterns informs our understanding of how genes influence lethality. This Editorial aims to highlight ways in which the *lacZ* expression profiles can impact on biomedical research by uncovering as-yet-unknown genotype-phenotype relationships, and through predicting the role of genes in health and disease.

## Abstract (precis)

**Summary:** A recent report by White et al. highlights the utility of *lacZ* reporter gene expression profiling as a powerful means of elucidating the role of genes in health and disease.

## *In situ lacZ* gene expression profiling: the missing link in understanding genotype-phenotype relationships

The Persian scholar Avicenna (980-1037) is credited with pioneering the idea of a syndrome in which symptoms affecting multiple organ systems can appear concurrently in a single patient (Abu-Asab et al., 2013). Such syndromes are an example of genetic disease, and are a hot topic in biomedical research because there is the hope that an understanding of the genetic predisposition to ‘phenotypic’ disease processes affecting one or more organ systems could pave the way for better diagnosis and better treatment of a range of disorders. However, there are gaps in our current understanding of genotype-phenotype relationships. For example, for most genes in the mouse and human genomes, functional annotation can only be predicted, with only 7872 genes of a potential 24,190 protein-coding genes in the mouse being annotated by the Gene Ontology (GO) consortium in terms of biological function (Blake et al., 2015). In addition, from a disease perspective it is important to know whether a gene involved in a respiratory process, for example, is expressed in the bronchi, the bronchioles or the alveoli, necessitating a high level of granularity in annotation. In many cDNA microarray studies, researchers were content to homogenise tissue in an effort to isolate total RNA prior to expression profiling; however, detailed granularity was often lost using this method. Furthermore, there is the thorny issue of genetic redundancy, i.e. another gene-family member can compensate for the lack of a functional gene. The latter issue requires a detailed knowledge of co-expression at the tissue subcompartment level, which is also difficult to glean from studies of homogenised tissue. As a means of addressing these problems, in this issue of Disease Models & Mechanisms (DMM), Jacqueline White and colleagues report on recent work accomplished by the Wellcome Trust Sanger Institute in characterising the *in situ lacZ* expression profiles of 424 genes in the adult mouse (Tuck et al., 2015). By using a *lacZ* reporter gene, which encodes a β-galactosidase enzyme that, in the presence of an X-Gal substrate, stains tissues blue, White and colleagues have annotated whole-mount *in situ* gene expression patterns in a comprehensive range of up to 47 organ systems in the adult mouse. This gene expression dataset is closely linked to the phenotyping effort of the International Mouse Phenotyping Consortium (IMPC), which is a major international effort to explore the effects of knocking out 20,000 genes in the mouse. Thus far, the IMPC have generated phenotype data for 2469 knockout lines (http://www.mousephenotype.org/), and the 424 gene expression patterns that have been annotated and analysed by White et al. represent an important attempt at pooling together expression data and correlating this with genotype and phenotype.

To understand how a detailed knowledge of gene expression will help researchers elucidate genotype-phenotype relationships, one has to understand that the risk factors associated with many human disease conditions are complex and include lifestyle choices, environment and multiple genetic loci. Consequently, it is not always clear to researchers investigating human disease as to why a polymorphism or a mutation at an individual locus can predispose an individual to a disease condition. Exploring the expression pattern of a candidate gene in a mouse model can provide insight into the molecular mechanisms involved. To highlight one of the examples discussed by White et al., the *Apoo* gene, which encodes the mitochondrial protein apolipoprotein O, has recently been implicated in lipid accumulation and diabetes-related heart disease and is shown to be expressed in the heart and pancreas of *Apoo lacZ* reporter mice. It is possible that this mitochondrial protein represents an important link between heart disease and pancreas dysfunction. In line with this, it is known that fatty-acid-challenged cardiomyocytes have a tendency to remodel mitochondrial structure and function (Elezaby et al., 2015), although the genetic basis of this is poorly understood. Furthermore, from human studies it is known that ectopic fat deposition occurs in cases of endocrine and exocrine pancreas dysfunction (Gaborit et al., 2015), yet the metabolic pathways that regulate lipid accumulation in these patients remain unclear. The expression pattern of apolipoprotein O highlights a tantalising link between these two organ systems and, by using the *lacZ* gene expression profiles in this way, researchers are able to tap into this resource to generate hypotheses and explore primary associations between gene and tissue type.

## Delving deeper: expression information at the subcompartment level

Expression profiles that detail gene expression in multiple tissue subcompartments are of great interest to the pathology community, biomedical researchers and to clinicians. Of great significance, the authors annotated gene expression patterns at a sufficient granularity to help elucidate the mechanisms of tissue injury and repair. For example, the authors report on the striking expression pattern of *Ldhb*, which stains the bronchi and bronchioles, but not the alveoli, of the mouse lung. *Ldhb* encodes lactate dehydrogenase B, an enzyme of the glycolytic pathway involved in the conversion of pyruvate to lactate. The pattern of *Ldhb* expression could provide insight into degenerative conditions such as centriacinar emphysema, which targets the terminal bronchiole, and could enable a mechanistic understanding of why this differs from panacinar emphysema, a degenerative condition of the alveolar sacs. In the latter example, compartmentalisation along the proximodistal axis was particularly relevant. Subcompartment-specific expression patterns highlight key differences between anatomical substructures and have the potential to provide insight into disease mechanisms. For example, in the nephron, it is known that the proximal convoluted tubule in the cortex of the kidney is particularly susceptible to damage from ischaemia-reperfusion injury (Liu et al., 2014). The reasons for this are currently unclear but are of great clinical significance because the mechanisms of this process could allow for informed decisions on appropriate treatments that could help to limit the transition from acute kidney injury (AKI) to chronic kidney disease (CKD). By offering a web resource that has the potential to uncover genes that are expressed in, for example, the proximal convoluted tubule, but not the loop of Henle, distal tubule or renal corpuscle, pathways that underlie pathogenesis and represent new therapeutic candidates could be pinpointed. This feature should also be of great relevance to the stem cell research community, who could use the *lacZ* expression profiles to identify the various stem cell niches that exist in the adult mouse. For some organ systems, such as hair follicles (Jaks et al., 2008; Snippert et al., 2010), stomach (Barker et al., 2010), small intestine and colon (Barker et al., 2007), master regulatory genes that define the stem cell population are known, but, in most organ systems, the expression profile of the stem cell niche has still to be elucidated. The *lacZ* gene expression patterns and supporting annotation might allow stem cell researchers to explore the gene expression profiles of *in vivo* stem cell compartments in greater depth. This is potentially important from a disease perspective because subtle changes in the complexity of the stem cell niche might underlie changes in proliferation, differentiation and morphology that are observed in various pathological conditions. It is additionally important in providing an *in vivo* profile of stem cell niches in terms of marker gene expression that can be compared to patient-derived organoid systems, which are simple experimental systems devoid of blood and immune cells that are increasingly used to correlate gene and function in human studies.

Furthermore, in addition to screening gene expression in reproductive, musculoskeletal, digestive, nervous, cardiovascular, respiratory, glandular and sensory systems, the authors provide annotation of expression in key organs of the immune system, including spleen, Peyer's patches and mesenteric lymph nodes. The latter observations are very important because it is often unclear in a disease condition whether an abnormal gene expression profile represents, for example, an injury response of the tissue parenchymal cells that permanently ‘reside’ in the tissue, or whether they are related to an invasion of ‘migratory’ immune cells, such as macrophages, neutrophils or natural killer (NK) cells, into the damaged tissue. Annotation of the immune cell compartment of the various organ systems is often overlooked in gene expression screens, and by delivering annotation in this way the authors are generating a resource of wide interest to the biomedical community.

## *lacZ*-omics as a means of exploring genetic redundancy and lethality

The authors additionally report on their initial analysis of the 424 *lacZ in situ* expression patterns. Enrichment analysis reveals that most genes are commonly expressed both in testes and brain, whereas adipose tissue and mammary gland show highly restricted patterns of gene expression. It will be interesting to compare the annotated expression patterns of testes and brain with the *in situ* hybridisation expression patterns generated by the GUDMAP consortium (genitourinary development; www.gudmap.org) and the Allen Brain Atlas (http://www.brain-map.org/) to ensure that they validate one another. Importantly, White et al. included longitudinal whole-mount sections of brain in their follow-up survey of exploring subcompartment localisation of 125 *lacZ* expression patterns, and these are directly comparable to the Allen Brain Atlas sagittal section *in situ* hybridisation dataset. In the mammary gland, where a restricted expression pattern was observed, it would be interesting to explore the dynamic nature of gene expression of this small number of genes in ductal branching morphogenesis, alveolar bud formation, lactation and involution because this might reveal novel insights into mammary gland development in the adult.

Another interesting and highly significant observation is the association between gene expression and lethality. Homozygous viability was captured during the IMPC phenotyping screen and, when compared to the gene expression profiles of *lacZ* reporter mice, it was shown that the genes causing lethality when inactivated were more likely to be expressed in a large number of tissues. Furthermore, by using GO term enrichment, the authors provide evidence that lethal and subviable genes were more likely to be involved in a greater number of biological processes. Clearly, an interesting pattern is developing; however, once the *lacZ* reporter expression screen extends to include *all* members of a gene family it will be possible to explore whether lethality correlates, or indeed anti-correlates, with genetic redundancy. In this scenario, one could envisage that it is ultimately the uniqueness of an expression pattern, and therefore the lack of a paralogue to compensate for the deleted gene's function, that is the crucial determinant of embryonic and/or perinatal lethality.

In future studies, it will be interesting to explore whether a multivariate analysis that includes phenotype associations and a measure of co-expression per organ system will offer a refined understanding of the role of genes in tissue compartments and subcompartments. To this end, an analytical tool is envisaged that would allow researchers to assess the likelihood of a knockout displaying a phenotype – or perhaps even lethality – in a given tissue. By virtue of the >1 million phenotyping assays made freely available on the IMPC resource, this is a testable hypothesis and would offer one method of refining genotype-phenotype associations to the long-awaited position in which they can begin to offer accurate predictions. A detailed and comprehensive understanding of gene co-expression at the tissue subcompartment level is central to this analytical approach and, as more *lacZ* reporter expression patterns are made freely available by the Wellcome Trust Sanger Institute, the means of predicting the role of genes in health and disease will become a reality.
